# Exploring the Esoteric: Tuberculosis of the Medial Epicondyle of the Humerus

**DOI:** 10.7759/cureus.44610

**Published:** 2023-09-03

**Authors:** Prasad Soraganvi, Pothuri R Ram, Harsha Vardhan Alle N Reddy, Madhan Jeyaraman, Sankalp Yadav

**Affiliations:** 1 Orthopaedics and Trauma, PES Institute of Medical Sciences and Research, Kuppam, IND; 2 Orthopaedics and Trauma, Sanjay Gandhi Institute of Trauma and Orthopaedics, Bengaluru, IND; 3 Orthopaedics, ACS Medical College and Hospital, Dr. MGR Educational and Research Institute, Chennai, IND; 4 Medicine, Shri Madan Lal Khurana Chest Clinic, New Delhi, IND

**Keywords:** tuberculosis, bone tb, medial epicondyle, humerus, mtb (mycobacterium tuberculosis)

## Abstract

Tuberculosis (TB) of the medial epicondyle of the humerus represents a rare etiology of chronic pain, swelling, and restricted mobility of the elbow joint, characterized by a gradual progression of the swelling. The paucity of reported cases in the literature attests to their infrequency. Herein, we present a case that was referred to our department after enduring swelling, restricted movement, and discomfort for a period of one year. The patient underwent a thorough clinical evaluation, followed by debridement, which ultimately resulted in the diagnosis of TB of the medial epicondyle of the humerus. In this ensuing case report, we comprehensively describe the clinical presentation, diagnosis, and treatment approach employed for this uncommon manifestation of TB affecting the elbow joint.

## Introduction

Tuberculosis (TB) remains a pressing health concern in India, with a high prevalence and the potential to affect any organ system [1-3]. While pulmonary involvement is the most common manifestation, skeletal TB can also occur, leading to joint destruction and deformity. Among the upper limb joints, the elbow is frequently affected, with reported incidence rates ranging from 2% to 5% [4-6]. Patients with elbow TB may present with joint pain, swelling, limited range of motion, and functional impairment. Although the diagnosis is primarily based on imaging studies and polymerase chain reaction (PCR) analysis, prompt identification and treatment are crucial for preventing disease progression and minimizing disability. Here, we present a rare case of TB involving the medial epicondyle of the humerus, highlighting the clinical features, diagnostic workup, and management approach for this uncommon presentation. Our report underscores the importance of considering TB in the differential diagnosis of elbow pathology, especially in regions with a high burden of the disease.

## Case presentation

**Figure 1:**
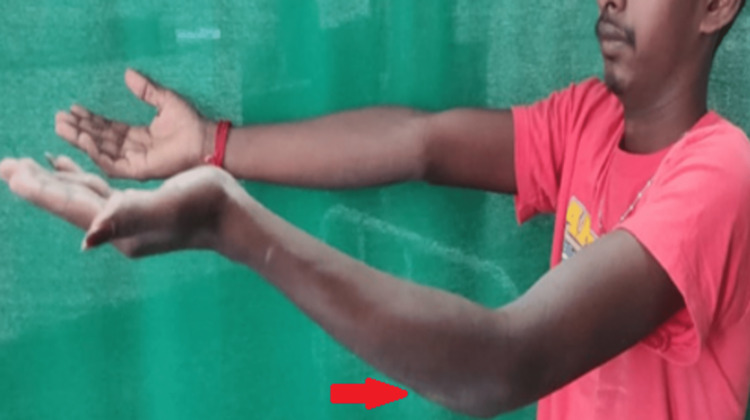
Gross image showing left elbow swelling and flexion deformity

We present the case of a 21-year-old male patient who presented with a complaint of left elbow joint swelling, pain, and deformity persisting for two and a half years. Upon physical examination, a 4x4 cm swelling was observed on the medial side of the right elbow joint, along with a flexion deformity, mild tenderness, and restricted movements (Figure 1).

**Figure 2:**
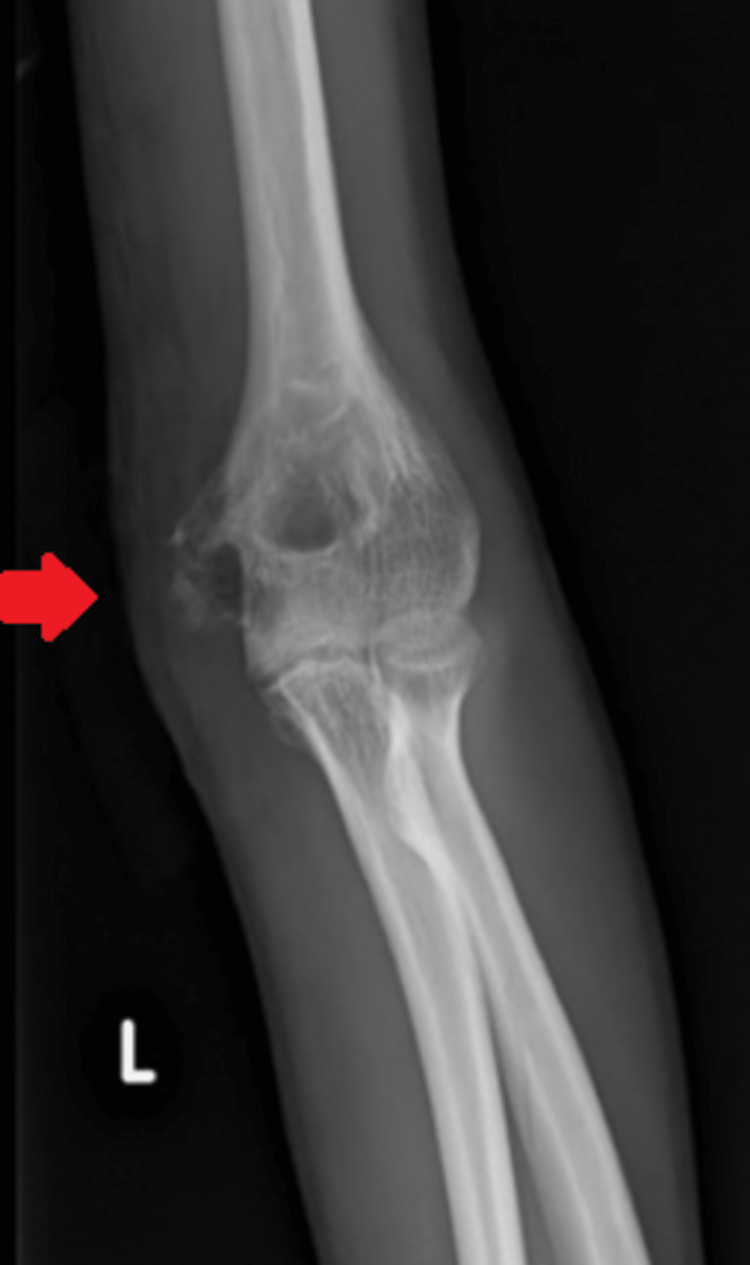
Anteroposterior X-ray of the left elbow joint Arrow showing lytic lesions in the medial epicondyle of the left humerus

**Figure 3:**
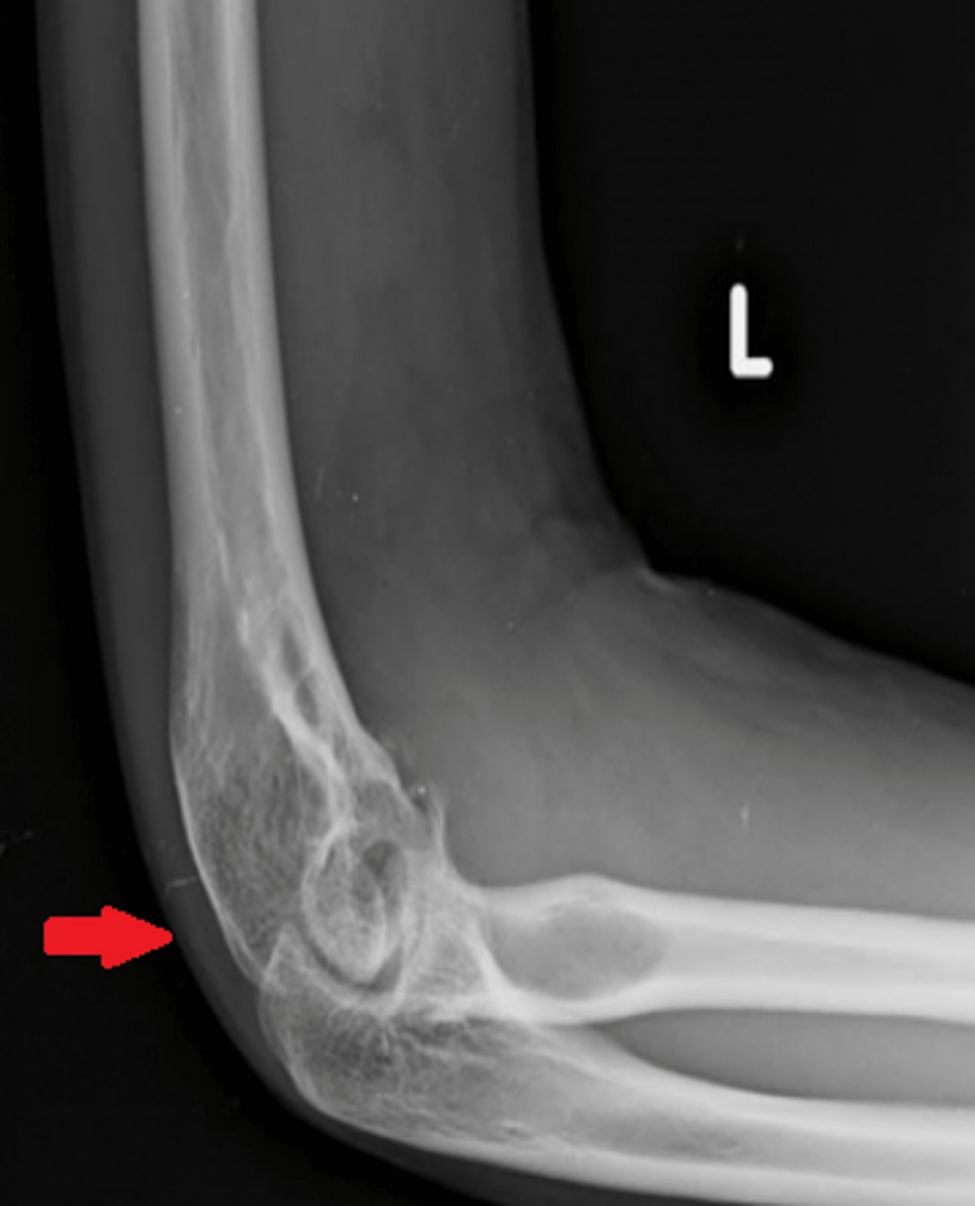
Lateral X-ray of the left elbow joint Arrow showing lytic lesions in the medial epicondyle of the left humerus

Plain X-rays revealed a lytic lesion in the medial epicondyle of the left humerus (Figures 2, 3).

**Figure 4:**
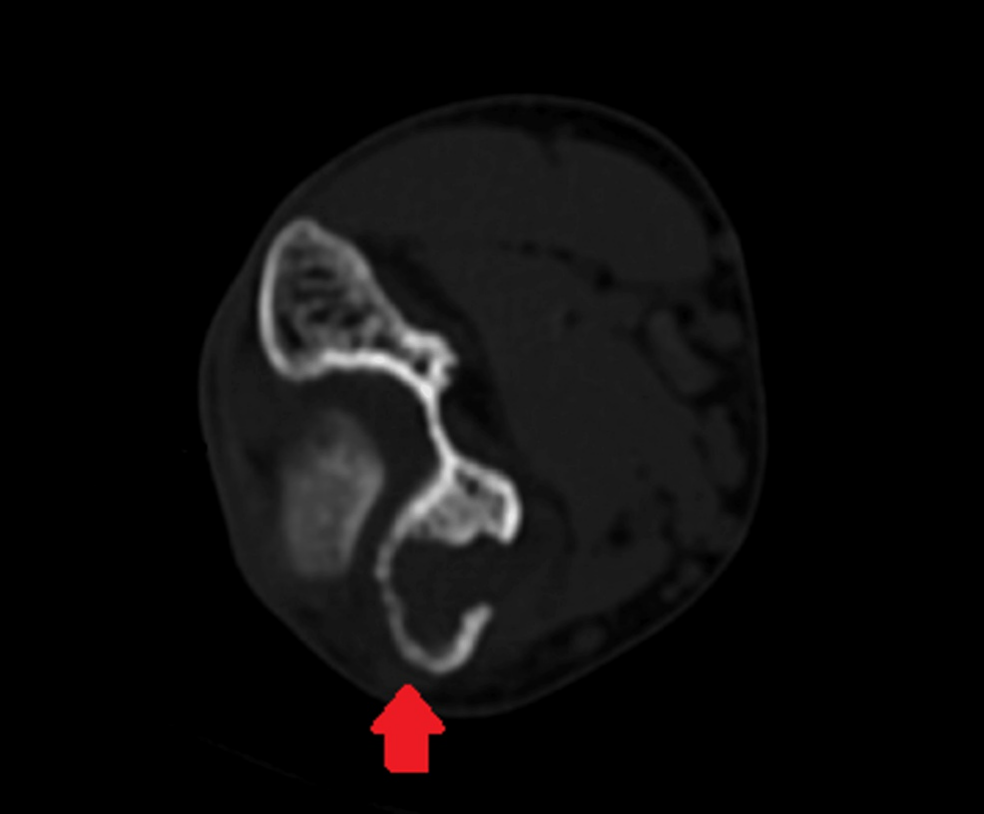
CT scan of the left elbow joint Arrow showing a lytic lesion involving the medial epicondyle of the humerus, cortical erosions, and adjacent soft tissue extension CT: computed tomography

This prompted a computed tomography (CT) scan, which reported a lytic lesion involving the medial epicondyle of the humerus, cortical erosions, adjacent soft tissue extension, and joint effusion (Figure 4).

**Figure 5:**
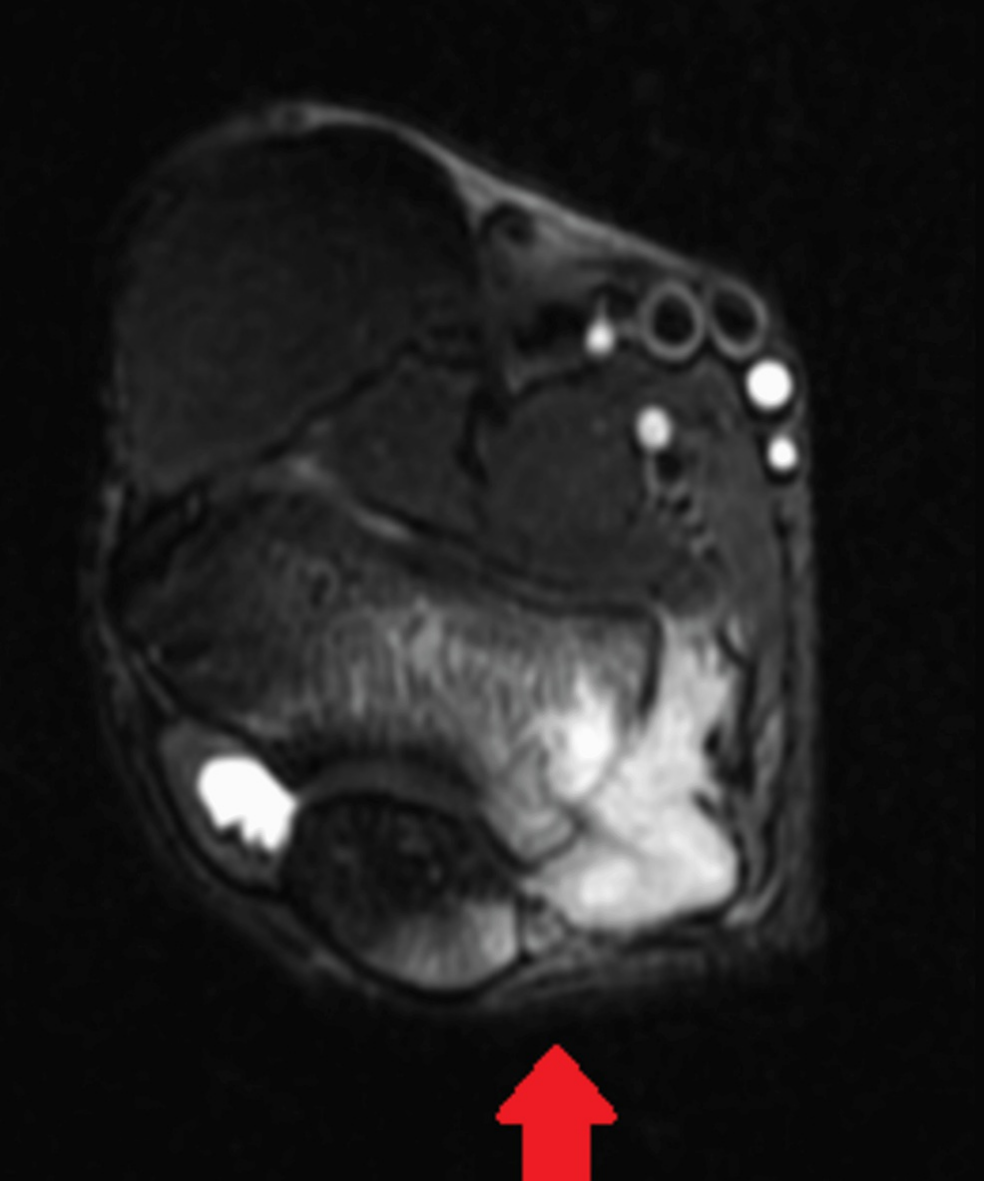
MRI of the left elbow joint Arrow showing a T2-weighted image with involvement of the medial epicondyle of the left humerus MRI: magnetic resonance imaging

**Figure 6:**
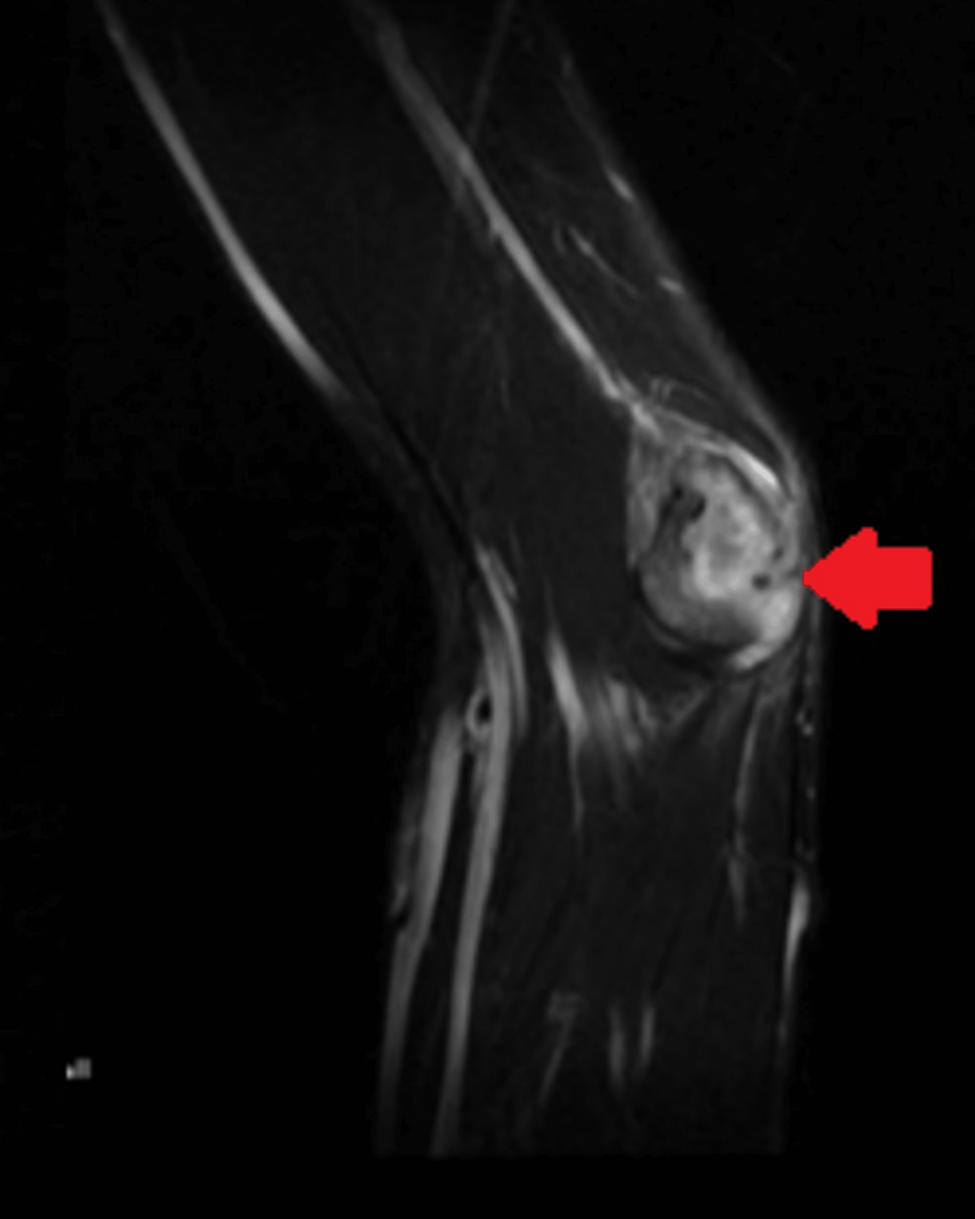
MRI of the left elbow joint Arrow showing a T1-weighted image with involvement of the left humerus MRI: magnetic resonance imaging

The magnetic resonance imaging (MRI) of the left elbow joint was performed on a 1.5 Tesla MRI system using the standard protocol. The study revealed a focal lesion involving the medial epicondyle of the left humerus, measuring approximately 2.5 x 1.5 cm in size. The lesion showed T1 hypointense and T2 hyperintense signal intensities. The surrounding soft tissues demonstrated an increased signal intensity on T2-weighted images, suggesting edema and inflammation. The cortex of the medial epicondyle showed cortical thinning and erosion. There was associated soft tissue extension and joint effusion. The ulnohumeral and radiohumeral joints appeared unremarkable. The visualized musculature, tendons, and ligaments were unremarkable. There was no evidence of lymphadenopathy (Figures 5, 6).

His chest radiograph was unremarkable. An induced sputum for fluorescent microscopy and cartridge-based nucleic acid amplification tests of induced sputum were negative. Further, his HIV (I and II) were non-reactive. However, he had elevated erythrocyte sedimentation rate (ESR) and C-reactive protein (CRP) levels, which are non-specific markers of inflammation. The combination of elevated ESR and CRP levels, along with radiological findings, raised the suspicion of a chronic infectious process. Furthermore, routine investigations were done, including a pre-anesthetic evaluation. Subsequently, the patient underwent debridement, during which a sample was collected and sent for PCR analysis. The report confirmed the diagnosis of TB in the medial epicondyle of the left humerus.

**Figure 7:**
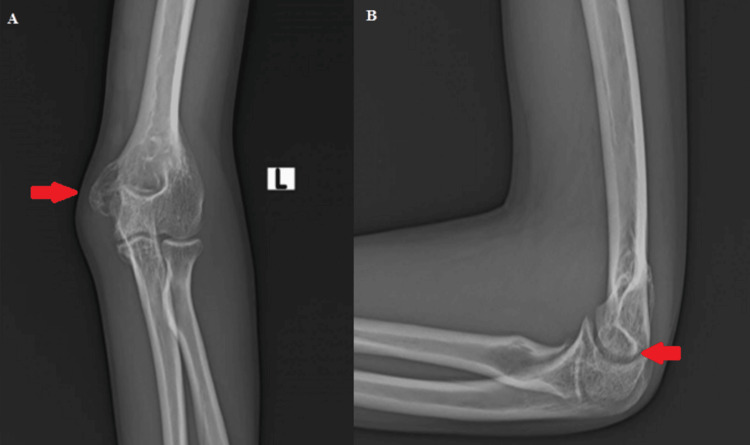
Postoperative X-rays of the left elbow joint were taken on the second day to assess the extent of debridement A: anteroposterior view; B: lateral view

Postoperatively, a repeat radiograph was taken on the second day to assess the extent of debridement, which demonstrated adequate debridement of the lesion (Figure 7).

**Figure 8:**
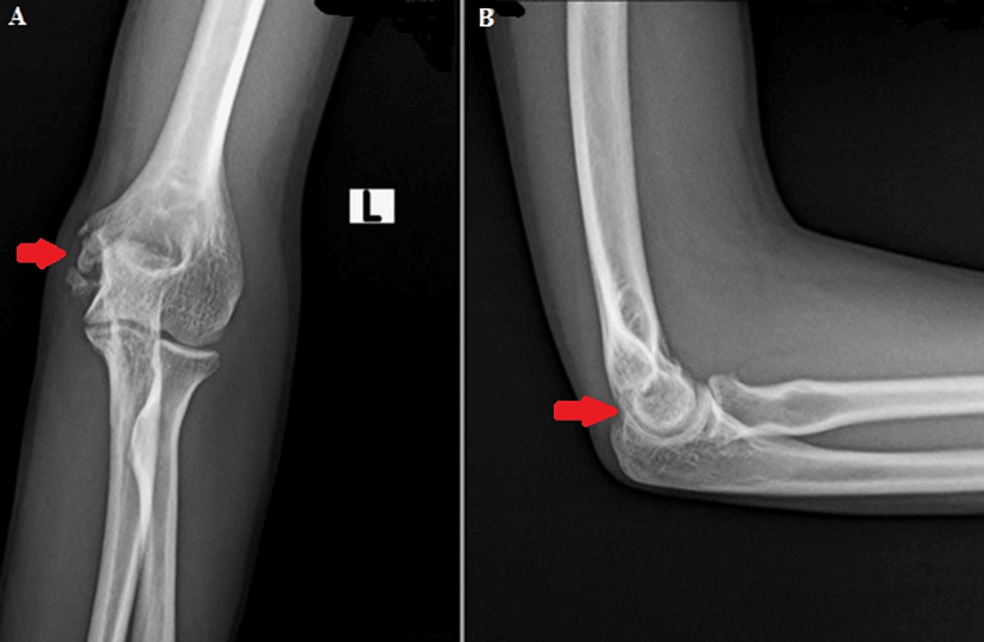
X-ray of the left elbow joint at one year of follow-up showing no signs of disease recurrence A: anteroposterior view; B: lateral view

He was initiated on anti-tubercular chemotherapy with fixed-dose combinations of isoniazid, rifampicin, pyrazinamide, and ethambutol per the national guidelines for 12 months. At one year of follow-up, the patient's range of motion had returned to normal, and a follow-up radiograph showed no signs of disease recurrence (Figure 8).

Our case underscores the importance of the early diagnosis and appropriate treatment of TB involving the medial epicondyle of the humerus to prevent joint destruction and functional impairment.

## Discussion

TB affecting the musculoskeletal system is a relatively uncommon medical condition, constituting only 5% of such infections [7-9]. Within this category, tuberculosis in the elbow joint is a rare occurrence, accounting for 5% of musculoskeletal TB cases [10,11]. Tuberculous arthritis typically affects a single joint, although multiple joint involvement may be observed in 10% of cases. The disease can spread to the musculoskeletal system through various routes, including hematogenous, lymphatic, or direct local spread of tubercle bacilli from other lesions.

Symptoms of TB arthritis can be vague and elusive, presenting as local swelling, deformity, and pain in a single joint that persists for a prolonged period, accompanied by a gradual reduction in range of motion [12]. Joint swelling and pain that worsens with physical activity are its most common manifestations [8]. Due to the insidious progression of the disease and the subtle nature of its symptoms, diagnostic evaluations are often delayed until the disease has advanced significantly [13].

Tuberculous arthritis, in its early stages, can be easily mistaken for other conditions such as trauma, septic arthritis, or rheumatoid arthritis. Unfortunately, there is often a significant delay between the onset of symptoms and a definitive diagnosis, with delays ranging from five to 47 months [14,15]. While systemic symptoms, such as low-grade fever, fatigue, decreased appetite, weight loss, night chills, tachycardia, and anemia, may occur, they are not commonly observed. In the case presented herein, the patient reported pain and swelling in the left elbow joint. Suspecting septic arthritis or rheumatoid arthritis, various diagnostic investigations were conducted, ultimately revealing the presence of infectious arthritis. A debridement procedure was planned accordingly.

The constant elevation of CRP and ESR levels led us to suspect rheumatoid arthritis in this case. Given the absence of systemic or local signs of infection, we ruled out the chances of septic arthritis. While cases in which rheumatic fever is negative and unilateral involvement of the elbow and shoulder joints is observed are atypical, rheumatoid arthritis has a wide range of clinical variants. Even in cases where diagnostic criteria do not meet the guidelines proposed by the American Academy of Rheumatoid Arthritis, a tentative diagnosis of seronegative rheumatoid arthritis can still be made.

Imaging findings, such as osteolytic lesions, joint effusion, and peripheral enhancement of synovium on MRI, can help support the diagnosis of musculoskeletal TB. The gold standard for the diagnosis of musculoskeletal TB is to identify *Mycobacterium tuberculosis* through culture or PCR testing. However, these tests may have limited sensitivity, especially in cases with a low bacterial load or paucibacillary disease. In some cases, a diagnosis of musculoskeletal TB can be made based on the histopathological examination of the biopsy specimen, which may show granulomatous inflammation.

The treatment of musculoskeletal TB involves a combination of anti-tubercular drugs and surgical debridement if necessary. The duration of anti-tubercular chemotherapy is typically 12 months, depending on the extent and severity of the disease. It is important to monitor patients closely for treatment response and potential adverse effects of anti-tubercular drugs. As shown in the current case, a high level of suspicion is required to make an accurate diagnosis [16,17]. The incidence of musculoskeletal TB has been decreasing globally due to improved socioeconomic conditions and the use of anti-TB drugs, but it remains prevalent in certain regions, especially in developing countries.

## Conclusions

The diagnosis and management of musculoskeletal TB can be challenging due to its nonspecific clinical manifestations and imaging findings. A high level of suspicion is required, and a multidisciplinary approach involving infectious disease specialists, radiologists, and orthopedic surgeons is often necessary for optimal patient care. Early diagnosis and treatment are essential for favorable treatment outcomes and to prevent complications such as joint destruction and neurological deficits.
